# Retrospective Long-Term Survival Rate and Clinical Performance of Zirconium Oxide Restorations over the Past 5 Years: A Comparative Study Between Single Crowns and Fixed Dental Prostheses

**DOI:** 10.3390/medicina61020210

**Published:** 2025-01-24

**Authors:** Dan Lolos, Sorin Gheorghe Mihali, Stefania Dinu, Mihai Mitariu, Anca Tudor, Roxana Oancea

**Affiliations:** 1 Faculty of Dental Medicine, “Victor Babeș” University of Medicine and Pharmacy Timișoara, Eftimie Murgu Sq. No. 2, 300041 Timișoara, Romania; lolosdan@umft.ro; 2 Department of Prosthodontics, Faculty of Dentistry, “Vasile Goldis” Western University of Arad, 94 Revolutiei Blvd., 310025 Arad, Romania; 3 Department of Pedodontics, Faculty of Dental Medicine and Pharmacy, Victor Babes University of Medicine and Pharmacy, 9 No., Revolutiei 1989 Bv., 300041 Timișoara, Romania; dinu.stefania@umft.ro; 4 Department IV of Dental Medicine and Nursing, Faculty of Dentistry, University of Sibiu “Lucian Blaga” (ULBS), Lucian Blaga 2A, 550169 Sibiu, Romania; mihai.mitariu@ulbsibiu.ro; 5 Medical Informatics and Biostatistics, Research Center in Dental Medicine Using Conventional and Alternative Technologies, Faculty of Dental Medicine, “Victor Babes” University of Medicine and Pharmacy, 2 Eftimie Murgu Square, 300041 Timișoara, Romania; atudor@umft.ro; 6 Department of Preventive and Community Dentistry, Faculty of Dental Medicine, “Victor Babeş” University of Medicine and Pharmacy, 300041 Timișoara, Romania; roancea@umft.ro; 7 Translational and Experimental Clinical Research Centre in Oral Health, Department of Preventive, Community Dentistry and Oral Health, University of Medicine and Pharmacy “Victor Babes”, 300040 Timișoara, Romania

**Keywords:** zirconium oxide restorations, survival rate, single crowns, fixed dental protheses

## Abstract

*Background and Objectives:* This five-year retrospective study evaluated the survival and clinical performance of 1143 zirconium oxide restorations, including both monolithic (144) and layered (999) restorations. *Materials and Methods:* The analysis included clinical records and follow-up data of patients treated with zirconium oxide restorations. Failures in layered restorations were examined, particularly focusing on chipping caused by unsupported feldspathic ceramic exceeding 1.5 mm. Monolithic restorations were used as a benchmark for durability. *Results:* The results demonstrated a high overall survival rate of 96.3%, with monolithic restorations achieving a perfect survival rate of 100%, while layered restorations had a survival rate of 95.8%. Failures in the layered restorations were primarily associated with chipping, especially when the unsupported feldspathic ceramic exceeded 1.5 mm. *Conclusions:* This finding highlights the importance of maintaining adequate support for the ceramic layer to prevent such complications. Monolithic restorations, in contrast, showed superior durability, with no failures reported, making them a more reliable option for long-term success. These findings emphasize the need for the careful selection of zirconium oxide restoration types based on the clinical context, particularly in cases where durability is critical.

## 1. Introduction

The introduction of zirconia as a material for dental restorations has revolutionized restorative dentistry. Known for its outstanding mechanical properties, biocompatibility, and improved esthetic outcomes, zirconia has been widely accepted in clinical practice. Zirconia restorations have been particularly praised for their exceptional flexural strength, ranging from 900 to 1200 MPa, making them suitable for both anterior and posterior restorations [1,2].

These restorations are especially favored for their ability to withstand occlusal forces, ensuring long-lasting durability in challenging clinical conditions.

Zirconia’s high strength and fracture resistance are critical factors in its use for single crowns and fixed dental prostheses (FDPs), where strength is essential for supporting functional demands [3,4]. Despite its remarkable strength, zirconia’s opaque nature has led to some limitations in anterior restorations, where esthetic demands require more translucency to mimic natural teeth.

To address this, layered zirconia restorations, which consist of a zirconia framework veneered with feldspathic ceramics, have been developed to enhance the visual appeal of the restoration, particularly in the anterior regions where esthetic properties are paramount [5,6]. However, these layered restorations are associated with potential complications, such as chipping and delamination of the porcelain veneer. Although zirconia is generally durable, the veneer layer is more susceptible to failure under certain conditions, raising concerns regarding the long-term clinical performance of these restorations [7,8].

Research comparing the survival rates and clinical outcomes of monolithic zirconia restorations (which use a single zirconia layer) and layered zirconia restorations (which include a veneering ceramic layer) is ongoing. While numerous studies highlight the exceptional durability of monolithic zirconia, with lower incidences of chipping and fracture, there remains debate about the relative performance of both configurations in terms of longevity, esthetic outcomes, and clinical complications [9,10]. Moreover, few studies have investigated the long-term survival rates of zirconia restorations when comparing single crowns and multi-unit fixed dental prostheses (FDPs). This gap in research is particularly significant, as multi-unit restorations often experience different functional stresses, which may influence the performance and longevity of zirconia materials in clinical settings [11,12].

Another aspect that has garnered attention is the durability of zirconia restorations when used in full-mouth rehabilitations or multi-unit FDPs. Although zirconia’s high fracture toughness has been noted as an advantage, concerns regarding the potential for veneer failure in multi-unit applications remain. The veneering ceramics can fracture or chip when subjected to occlusal forces, particularly in posterior regions, thus affecting the overall clinical success of layered zirconia restorations. These issues make it critical to assess the long-term clinical performance of zirconia restorations, with a focus on both single crowns and FDPs, to determine the most reliable restoration choice for various clinical situations [13,14].

A major concern in the field is the risk of chipping and delamination of the veneer layer in layered zirconia restorations. Previous studies have indicated that chipping is more likely to occur when the thickness of the feldspathic veneer exceeds certain thresholds or when restorations are subjected to excessive functional stresses. While monolithic zirconia restorations are not without their esthetic limitations, they do not present the same risk of veneer failure and are increasingly favored for posterior restorations where strength is paramount [15,16]. 

Even though both feldspathic ceramics and lithium disilicate-based ceramics offer aesthetic and minimally invasive advantages over the remaining dental hard tissue [17,18], they present lower structural resistance. Feldspathic ceramics exhibit flexural strength properties in the range of 62–90 MPa [19,20], while lithium disilicate falls within 397 MPa [21]. However, these two systems cannot be compared to the structural resistance offered by restorations made from zirconium or metal-ceramic. 

A comparison of study results reveals a difference of approximately 1000 N between restorations made from metal-ceramic (2587.08 N) and those made from zirconium (1361.00 N) [22], indicating that these two systems are in a higher category in terms of structural resistance. Zirconium (ZrO_2_) has a flexural strength of approximately 1000–1200 MPa when used in the form of high tetragonal ZrO_2_ [23,24]. Other studies suggest that the structural resistance of zirconium is somewhere between 900–1200 MPa [25,26]. Given these properties, zirconium can be classified as a material with superior resistance, standing out as the most resistant ceramic material. This denotes the fact that, although zirconium is considered both aesthetic [27] and mechanically strong [23,24,25,26,28], its opacity limits its use in various regions of the oral cavity [29].

Zirconia restorations are favored for their outstanding mechanical strength, biocompatibility, and esthetic flexibility, making them suitable for both anterior and posterior dental applications. The material is known for its high resistance to fracture, providing a reliable solution in dental restorations [30,31]. Although layered zirconia restorations enhance the esthetic appearance by incorporating a veneering ceramic, they may encounter issues like chipping and delamination, especially in multi-unit prostheses subjected to higher occlusal forces [32,33]. However the higher opacity of zirconia and metal-ceramic restorations limits their suitability for visible areas, where translucency is crucial for achieving a more natural, tooth-like appearance [34].

Therefore, the aim of this study is to evaluate and compare the long-term survival rates and clinical performance of zirconia restorations, focusing on single crowns and fixed dental prostheses (FDPs), over a five-year follow-up period. The study will examine both monolithic and layered zirconia restorations, comparing their mechanical properties, esthetic outcomes, and the incidence of complications such as chipping, fractures, or veneer delamination. This research seeks to provide more insight into the factors that influence the clinical outcomes of zirconia restorations and to clarify the advantages and disadvantages of both restoration types in different clinical scenarios.

### 1.1. Aims of the Study

This study aims to investigate the long-term survival rates and clinical performance of zirconia restorations. Specifically, it will compare single crowns and fixed dental prostheses (FDPs) over a five-year period. The key objectives include evaluating the mechanical strength, esthetic results, and the occurrence of complications such as chipping and fractures in both monolithic and layered zirconia restorations.

### 1.2. Null Hypotheses

It is hypothesized that there will be no significant difference in the long-term survival rates and clinical performance between single crowns and fixed dental prostheses (FDPs) made from monolithic or layered zirconia restorations.

## 2. Materials and Methods

This retrospective study involved 393 patients aged between 18 and 71 years, with a mean age of 37.5 years. Among the participants, 224 were women and 169 were men. The men exhibited a mean age of 38.7 years, slightly higher than the women’s mean age of 36.3 years. The patients sought treatment at our private dental clinic between 2013 and 2024, all under the care of the same clinician (S.G.M.), who used standardized clinical and laboratory protocols. Each restoration was evaluated after 5 years of function to be considered a prosthetic success, resulting in a total of 1144 zirconia restorations.

The study was conducted in accordance with the Declaration of Helsinki, and ap-proved by the Ethics Committee of the “Victor Babeș” University of Medicine and Pharmacy from Timisoara, Romania Nr. Nr. AV_06b/09.01.2019 rev 2024. All patients agreed to participate in the study and gave their written informed consent.

Monolithic zirconium oxide restorations were chosen for cases requiring high mechanical strength, particularly in posterior regions with significant occlusal loads. These restorations were fabricated using CAD/CAM systems, ensuring precise fit and uniformity. The monolithic structure minimizes the risk of chipping, as no feldspathic veneering is present. The decision to use monolithic restorations was guided by their demonstrated superior fracture resistance and wear compatibility with opposing dentition. Layered zirconium oxide restorations were selected for anterior regions where esthetic demands were higher. These restorations involved a zirconium oxide core veneered with feldspathic ceramic to enhance translucency and mimic natural enamel. The veneering process adhered to manufacturer guidelines, maintaining feldspathic ceramic thickness below the critical threshold of 1.5 mm to minimize the risk of chipping. Layered restorations were prioritized for their superior optical properties, particularly in cases requiring a high level of customization to achieve natural esthetics.

Our study investigated two different approaches to dental restorations utilizing zirconia oxide. The first approach focused on single-tooth restoration using zirconia oxide restorations, while the second approach centered on fixed prosthodontic restorations, which involved addressing multiple-tooth restorations. Within these approaches, two distinct techniques were employed: Monolithic zirconia restorations were fabricated from yttria-stabilized tetragonal zirconia polycrystal (Y-TZP) (BruxZir^®^ Zirconia, Glidewell Laboratories, Irvine, CA, USA), featuring a full-contour zirconia structure. The restorations were sintered at 1500 °C for optimal mechanical properties. Layered zirconia restorations used a Y-TZP substructure (Lava™ Zirconia, 3M ESPE, St. Paul, MN, USA), with feldspathic ceramic (Vita VM^®^ 9, VITA Zahnfabrik, Bad Säckingen, BW, Germany) layered over it. The layering process involved hand application and firing at 900 °C to achieve the desired esthetics and translucency. The zirconia frameworks were milled using a CAD/CAM system (PrograMill^®^, Ivoclar, Schaan, Liechtenstein) and adjusted as necessary before layering.

In our evaluation, the selection of zirconia restorations was based on a comprehensive assessment of factors including tooth fractures, failures of previous restorations, zirconia stratified previous restorations, or those made from materials with inferior strength compared to zirconia oxide, as well as losses of hard dental tissue. These considerations, coupled with tooth attrition, malocclusion, dental anomalies, and esthetic alterations, guided our decision-making process towards the appropriate treatment option.

The exclusion criteria were designed to ensure a focused and reliable study sample. Patients with systemic conditions or allergies affecting dental treatment, severe periodontal disease, active infections, or extensive caries requiring extractions were excluded. Additionally, individuals with severe bruxism, unrealistic treatment expectations, or significant dental trauma requiring complex care were omitted. Cases requiring unrelated surgical interventions, those unable to comply with follow-up, and patients with contraindications to local anesthesia or sedation were also excluded. Grouping these conditions enhanced clarity and maintained the study’s integrity.

**Figure 1:**
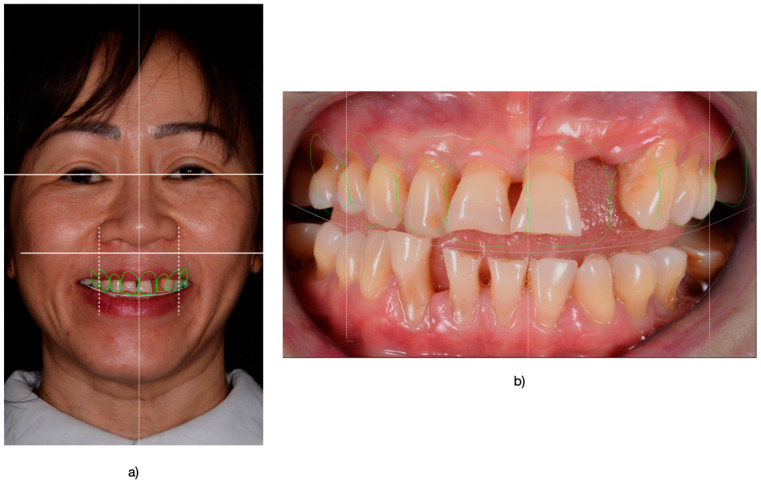
(**a**) Patient undergoing a comprehensive esthetic analysis, with a focus on evaluating facial proportions to ensure harmonious dental and facial relationships. (**b**) Intraoral view highlighting the detailed esthetic analysis of the upper arch, emphasizing the alignment and proportions critical for achieving optimal esthetic outcomes.

Initially, patients underwent an assessment (Figure 1) for both esthetic and functional aspects. Preliminary impressions were taken using alginate (Zhermack Hydrogum, Zhermack, Badia Polesine, VE, Italy), ensuring accurate initial models for further treatment planning. The models were made with type IV dental stone (GC Fujirock EP, Tokyo, Japan).

**Figure 2:**
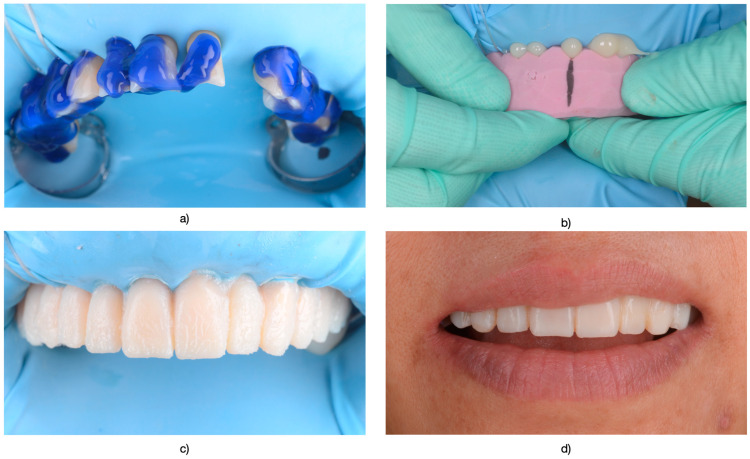
(**a**) Application of 36% orthophosphoric acid (Blue Etch, Cerkamed, Stalowa Wola, Poland) to prepare for bonding following the mock-up procedure. (**b**) Placement of the mock-up template in the oral cavity, with a midline marked for additional guidance during insertion. (**c**) Appearance of the mock-up (Protemp, Poland) immediately after insertion. (**d**) The mock-up underwent finishing procedures to eliminate irregularities and excess material, ensuring optimal contouring and esthetic refinement.

Subsequently, teeth requiring treatment underwent a diagnostic wax-up. This wax-up was then transferred into the patient’s mouth using a temporary resin material to create a direct mock-up (Protemp 4, 3M ESPE, Seefeld, Germany) (Figure 2). Following a 1–2 week testing period with the mock-up the decision to proceed with either full-contour (monolithic) zirconia or zirconia-supported restorations with layered feldspathic ceramic was determined based on occlusal parameters observed both on the articulator and after the testing period in the oral cavity using articulation paper (Arti-Fol^®^, Bausch, Lörrach, BW, Germany). Teeth were prepared with a chamfer margin and isolated using a rubber dam (OptraDam^^®^^ Plus, Ivoclar Vivadent, Schaan, Liechtenstein) to ensure a dry field during cementation.

**Figure 3:**
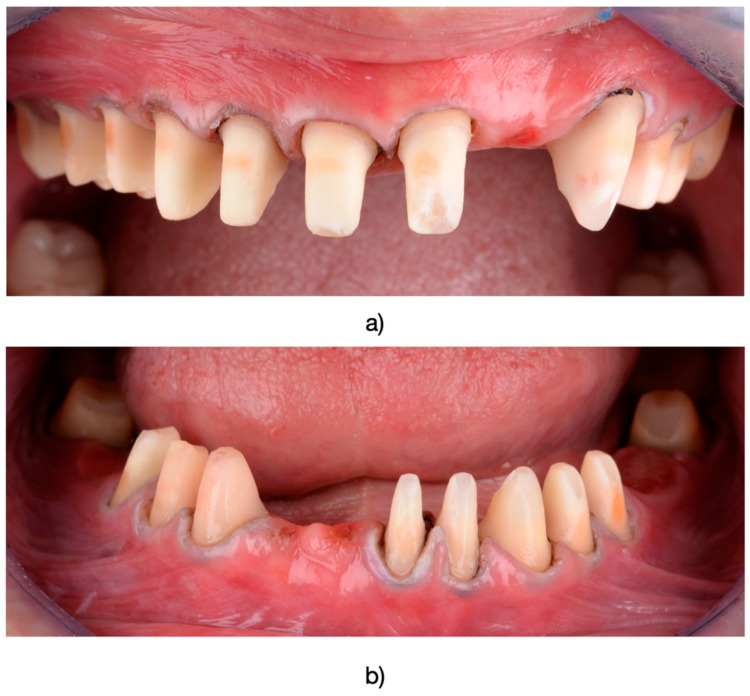
(**a**) Appearance after preparation of the upper arch. (**b**) Lower arch displaying prepared abutments. (Ultrapak, Ultradent Products, South Jordan, UT, USA) impregnated with a solution of 25% Aluminum Chloride (ViscoStat Clear, Ultradent Products, South Jordan, UT, USA) were used.

The dental preparations included reducing the occlusal and incisal surfaces to 2.0 mm, while the facial and lingual surfaces were reduced to 1.5 mm. The minimum abutment height was 5 mm, with an angulation degree ranging between 6° and 8° (Figure 3). Rounded or deep chamfer margins were finalized to ensure optimal integration of the restorations. In multi-unit restorations, necessary parallelism of preparations was maintained. The design of the copings adhered to a standard protocol, which included maintaining a minimum core thickness of 0.5 mm and a virtual cement layer thickness of approximately 40 µm. For posterior crowns, an occlusal veneer thickness of at least 1.0 mm was ensured. The veneer ceramic thickness was adjusted based on esthetic requirements and occlusal relationships for each individual patient.

The final impression was made with polyvinylsiloxane (Virtual 380, Ivoclar Viva-dent, Schaan, Liechtenstein) using a single-impression double-mixing technique with a standard tray after the mock-up was removed from the teeth for both arches. Immediate pouring followed with type IV dental stone (GC Fujirock EP, Tokyo, Japan). The color was recorded with a VITA classical shade guide (VITA Zahnfabrik, Bad Säckingen, Germany). Temporary restorations were fabricated to preserve tooth alignment, functionality, and esthetics, employing a mock-up technique as detailed in Figure 2.

In this study, the protocol for conditioning zirconium oxide restorations was consistently applied to both single crowns and fixed dental prostheses.

After the provisional restoration was removed, the prepared tooth surface was cleaned by sandblasting with 50 µm aluminum oxide particles at a distance of 10 mm for 10 s and subsequently de-greased using neophiline (Neophil, Ivoclar Vivadent, Schaan, Liechtenstein). Following degreasing, the tooth surface was thoroughly rinsed with water and then dried. The zirconium oxide restoration itself was conditioned by sandblasting with 50 µm aluminum oxide particles to increase surface roughness and promote mechanical retention. The abraded restoration surface was then decontaminated by applying 36% orthophosphoric acid (Blue Etch, Cerkamed, Stalowa Wola, Poland) for 1 min. After decontamination, the acid was rinsed off with water, and the surface was dried with air.

A bonding agent containing 10-MDP (Zir-Prime, BISCO, Schaumburg, IL, USA) was applied to the conditioned zirconium oxide surface and left to react for 1 min. The bonding agent was then air-thinned to ensure an even application. Finally, the conditioned and bonded zirconium oxide restoration were cemented using a dual-cure self-etch, self-adhesive resin cement (RelyX U200, 3M ESPE, Seefeld, BY, Germany). The cement was allowed to cure according to the manufacturer’s instructions, ensuring the optimal retention and stability of the restoration. This conditioning protocol was implemented uniformly across all zirconium oxide restorations evaluated in this comparative study, encompassing both single crowns and multi-unit restorations.

**Figure 4:**
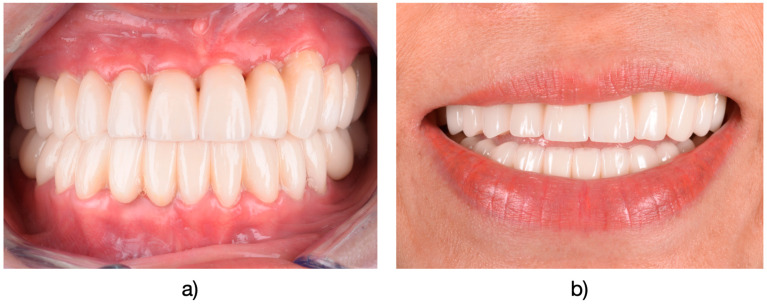
(**a**) Final restorations after intraoral fixation. (**b**) Final appearance of the restorations in the context of the patient’s smile.

**Figure 5:**
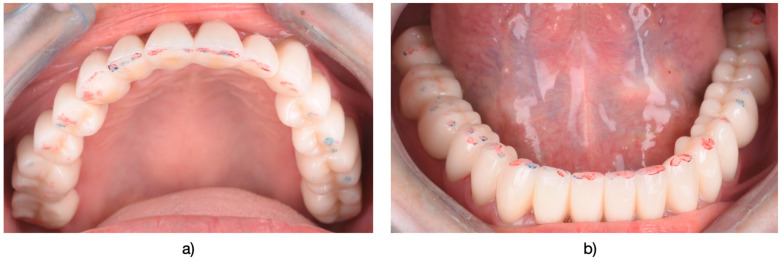
(**a**) Impressions left by the articulation paper following occlusal balancing in static (blue) and dynamic (red) conditions, ensuring multiple, equal, simultaneous, and balanced contacts (upper arch). (**b**) Similar impressions in the lower arch, showcasing the outcome of occlusal balancing.

The rubber dam was taken off, and any remaining occlusal adjustments were carried out in centric relation, as well as in lateral and protrusive movements. Following cementation (Figure 4), regular assessments were conducted every 6 to 12 months, coinciding with routine professional dental hygiene appointments. Final adjustments and finishing were performed using fine-grit diamond burs and polishing kits (OptraFine^®^, Ivoclar Vivadent, Schaan, Liechtenstein) (Figure 5). These assessments were performed by two dentists who were not involved in the initial restorative procedure at the time of cementation. For the conclusive reassessment, which was scheduled after a minimum of 5 years of functional use, all patients were recalled between January and June 2024.

**Figure 6:**
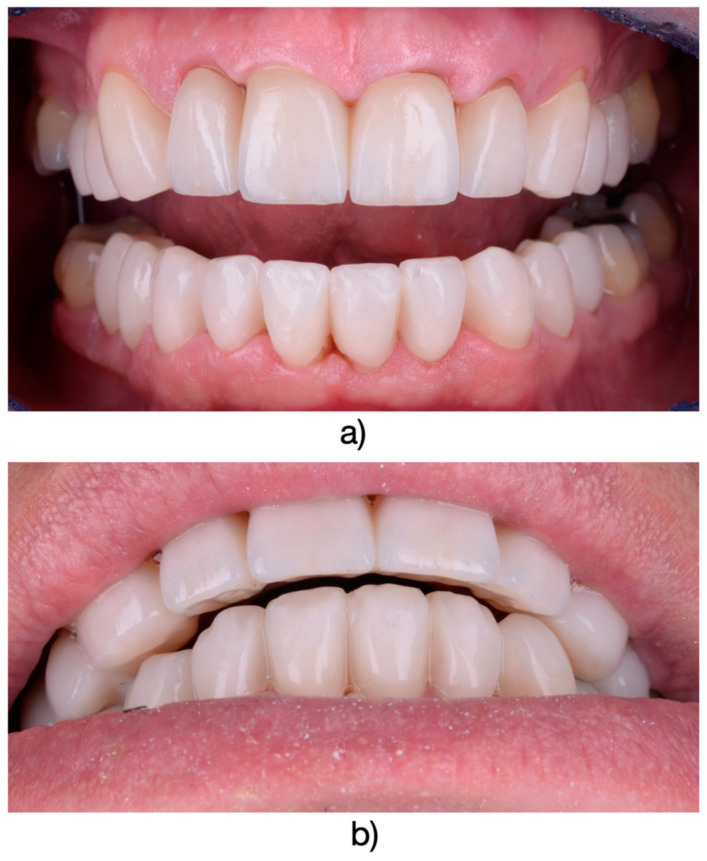
(**a**) Chipping is notably observed at teeth units 3.1, 3.2, and 4.1, 4.2, 4.3 (these instances of chipping manifest as missing fragments along the edges of restorations crafted using zirconium oxide-supported layered feldspathic ceramic). (**b**) Additionally, the image reveals irregularities on the vestibular surfaces of the affected teeth.

**Figure 7:**
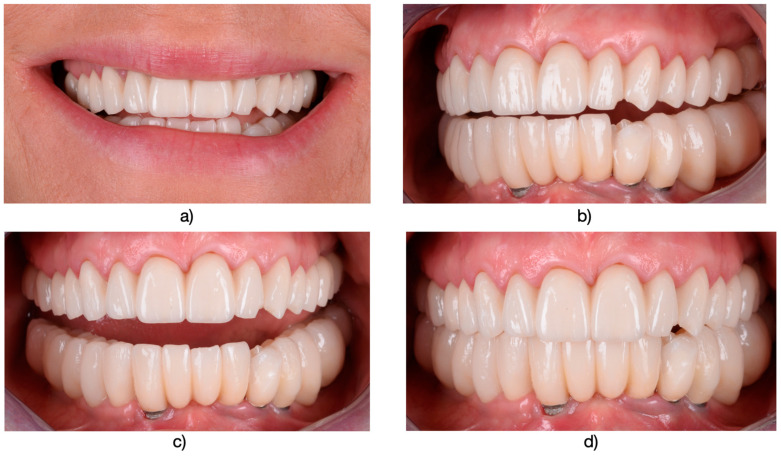
(**a**) The esthetic impact of the smile is significantly affected due to chipping observed on teeth units 2.2 and 2.3. (**b**) A semi-profile view highlights the chipping on these two teeth, providing a closer look at the extent of the damage. (**c**) The image also depicts the affected teeth in an endobuccal perspective, showcasing the spectrum of damage present. (**d**) It is noteworthy that this study will exclusively refer to the success rate of restorations on teeth with dental support. In this specific scenario, the restorations on the lower teeth were also fabricated on zirconium oxide support and subsequently layered. However, these restorations were placed on dental implants, highlighting a distinct treatment approach compared to restorations on natural teeth.

**Figure 8:**
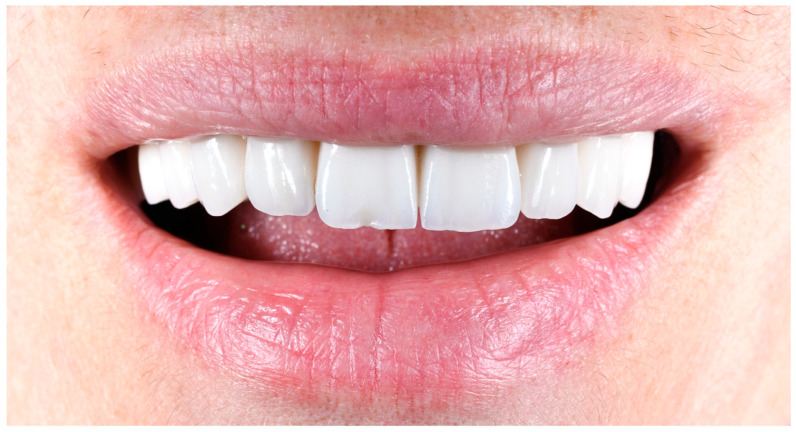
At dental unit 1.1, a noticeable chip can be observed. This restoration was fabricated on zirconium oxide support and subsequently layered with feldspathic ceramic. Despite diligent efforts to establish correct occlusal contacts, verified extensively on both articulated models and within the oral cavity, this failure still occurred.

**Figure 9:**
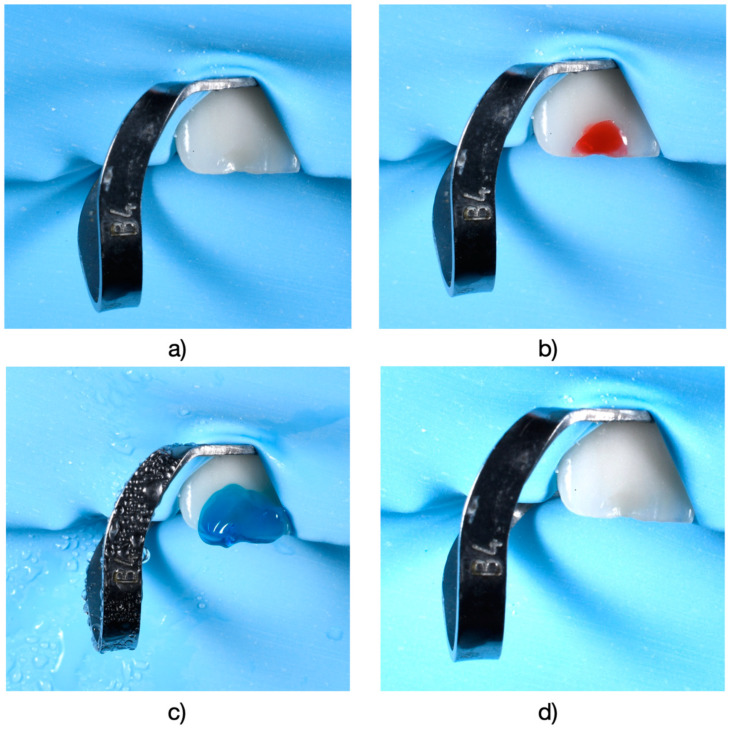
(**a**) A rubber dam was carefully placed, and the restoration undergoing chipping was prepared for conditioning. (**b**) Next, a solution of 3% to <7% hydrofluoric acid (IPS Ceramic Etching Gel, Ivoclar Vivadent, Schaan, Liechtenstein) was applied to the restoration surface and left for 60 s. Subsequently, the acid was thoroughly washed off with water and the area was dried. (**c**) Any crystalline debris or precipitate present on the ceramic surface was meticulously removed using 36% orthophosphoric acid (Blue Etch, Cerkamed, Stalowa Wola, Poland). (**d**) Following the removal of debris, the etched surfaces were then treated with a silane coupling agent (Monobond Plus, Ivoclar Vivadent, Schaan, Liechtenstein) for 60 s. After application, the surface was dried to achieve a monolayer of silane, ensuring optimal adhesion for subsequent procedures.

**Figure 10:**
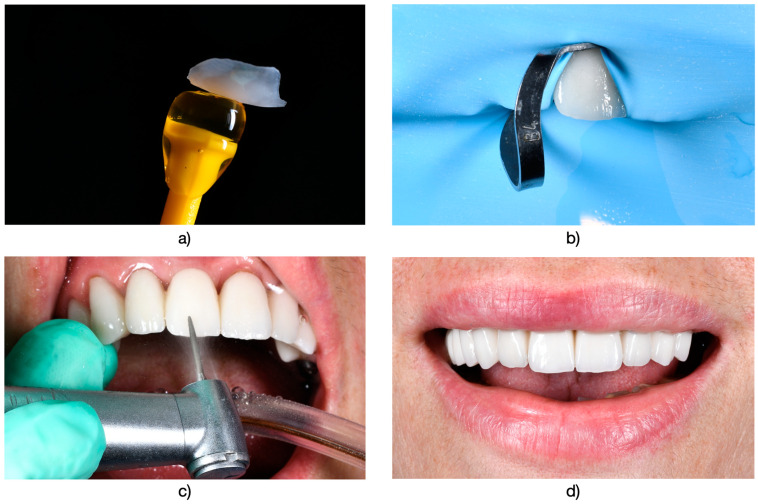
(**a**) Correction veneer crafted from feldspathic ceramic. (**b**) The veneer fixed onto the restoration using dual-cure luting resin cement (Variolink Esthetic LC, Ivoclar Vivadent, Schaan, Liechtenstein). Excess resin material was carefully removed using a microbrush (Ultrabrash, Dentalvision, Thailand) and hand instruments before polymerization. All surfaces were light-cured for 30 s at an intensity of approximately 1470 mW/cm^2^. (**c**) The incisal margin refined and polished post-cementation using red-stripe diamond burs (Sitea Romania) and OptaGloss diamond polishing rubbers (Ivoclar Vivadent, Liechtenstein). (**d**) Final appearance of the restoration after veneer fixation and finishing.

In this study, we comprehensively assessed the performance of zirconia-based dental restorations in relation to their interaction with antagonistic dentition. Our evaluation encompassed the analysis of occlusal force distribution and restoration margin wear using articulation paper (Arti-Fol^®^, XYZ Dental Products, USA). Additionally, we monitored occlusal compatibility and restoration adaptation based on clinically observed occlusal parameters. Chipping and its aesthetic implications, along with restoration and repair procedures, are demonstrated in Figure 6, Figure 7, Figure 8, Figure 9 and Figure 10. This approach enabled us to evaluate the impact of antagonistic teeth on restoration performance, providing pertinent insights for optimizing dental treatments.

Statistical analysis was conducted using JASP v0.17.1 software. Quantitative variables were presented as Mean ± Standard Deviation and Median (Quartile1-Quartile3). For differences based on the restorative material (layered or full contour), the type of restoration (single or multiple), and their positioning in the maxilla or mandible, we employed the Mann–Whitney U test. Categorical variables were described as number and percentage, and their associations were evaluated using the Chi-Square Test. To evaluate the overall differences in survival rates based on the type of restoration and their positioning in the mandible or maxilla, we made a Survival Analysis-Log Rank (Mantel–Cox) test with the SPSS v17 software. Results were considered significant for a value of *p* < 0.05.

## 3. Results

**Figure 11:**
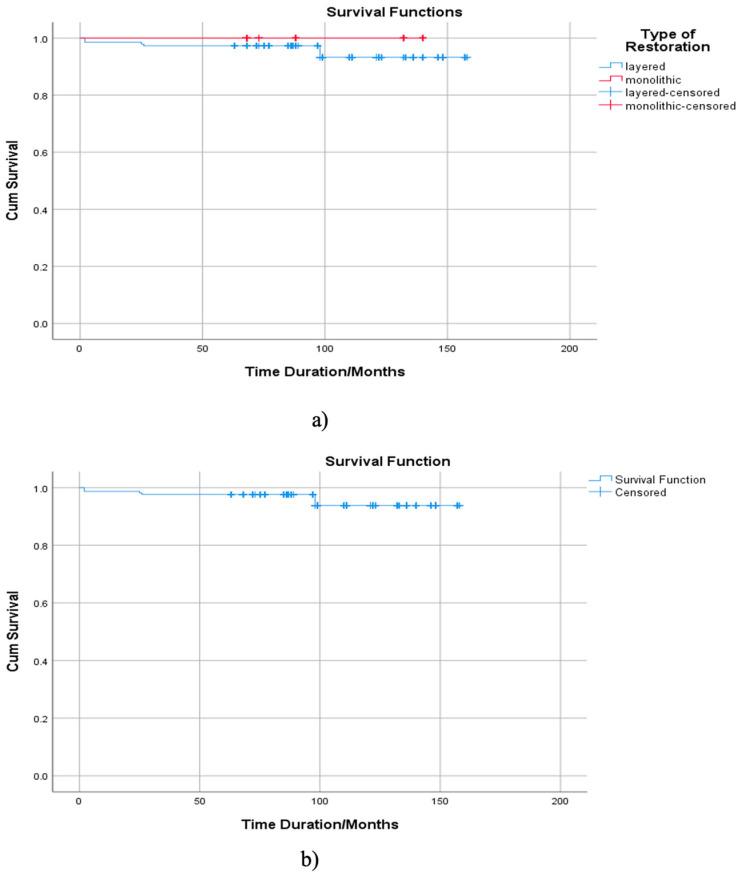
(**a**) Kaplan–Meier survival curve for zirconium oxide restorations, illustrating the survival rates for both monolithic and layered restorations over the study period. (**b**) Comparative survival period analysis between monolithic and layered zirconium oxide restorations.

A total of 1143 restorations were included in the study, comprising 144 monolithic and 999 layered restorations. Notably, there were 42 failures in the layered restorations and no failures in the monolithic restorations made from zirconium oxide. The overall survival rate was 96.3%. The mean survival period was 152.255 months (Figure 11). The survival rate for layered restorations was 95.8%, while monolithic restorations exhibited a 100% survival rate.

**Figure 12:**
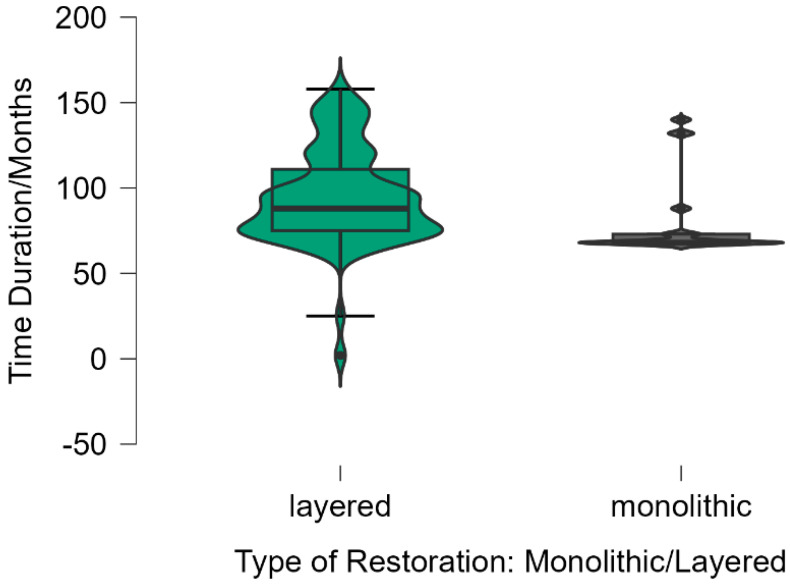
Superior Performance of Monolithic Restorations: A Comparative Study of Survival Duration (Months) Among 1143 Restorations.

The Kaplan–Meier analysis (Figure 11) confirmed significant differences between monolithic and layered restorations, showing a 100% survival rate for monolithic restorations compared to 95.8% for layered restorations. Figure 12 further highlights this statistically significant difference (Log Rank test, *p* = 0.027), with monolithic restorations demonstrating a markedly lower risk of failure (hazard ratio = 0.34, 95% CI: 0.12–0.95). Additionally, the comparative analysis illustrated in Figure 12 shows the superior performance of monolithic restorations, which exhibited no failures during the follow-up period.

The findings presented in Table 1 reflect the number of failure events and survival rates for each type of restoration. The 42 failures observed in layered restorations emphasize their increased susceptibility to failure, in contrast to the durability of monolithic restorations.

By comparing monolithic and layered zirconium oxide restorations, the Kaplan–Meier survival analysis revealed that monolithic restorations exhibited no failures throughout the study period, indicating superior long-term stability. In contrast, while layered restorations showed a high survival rate of 95.8%, 42 failures were recorded, with the majority due to chipping of the feldspathic ceramic layer (71.43%), followed by coronal and root fractures (14.29% each) (Figure 12).

The Log Rank (Mantel–Cox) test (*p* = 0.012) revealed that multi-tooth zirconium oxide restorations had significantly longer survival periods compared to single-tooth restorations (Table 2). In addition, restorations placed in the maxilla exhibited longer survival times than those in the mandible (Table 3).

The chipping rate for layered restorations increased progressively over time, with rates of 9.3% at 12 months, 14% at 24 months, and 30.2% at 36 months. Notably, no significant correlation was observed between failure rates and tooth position.

Table 4 presents the association between survival duration and restoration type. The mean survival duration for layered restorations was 94.29 ± 28.36 months, with a median of 88 months (range 75–111 months). In contrast, monolithic restorations had a mean survival duration of 79.71 ± 23.67 months, with a median of 68 months (range 68–73 months).

Table 5 summarizes the distribution of failure types in layered zirconium oxide restorations. The predominant cause of failure was chipping of the feldspathic ceramic layer (71.43%), followed by coronal fractures (14.29%) and combined coronal fractures with root fractures (14.29%). Despite these failures, the overall success rate remained high, as detailed below.

As seen in Table 5, the majority of failures (71.43%) were caused by chipping, consistent with the mechanical limitations of the feldspathic ceramic layer under occlusal forces. Coronal fractures and combined coronal fractures with root fractures each accounted for a smaller but equal proportion of failures (14.29%). Table 6 presents a comparative analysis of survival data for zirconium oxide restorations, evaluating differences between restoration types (monolithic vs. layered) and tooth position (maxilla vs. mandible, single tooth vs. multi-tooth).

The data in Table 6 indicate that single-tooth restorations exhibited significantly shorter survival durations compared to multi-tooth restorations, underscoring the greater stability of extended restorations. Similarly, maxillary restorations demonstrated superior survival times relative to mandibular restorations, likely due to biomechanical and functional differences between the two regions. These findings highlight the importance of considering tooth position and restoration type in clinical decision-making to optimize long-term outcomes.

Furthermore, the results emphasize the critical role of restoration type and tooth location in determining clinical success. While monolithic zirconium oxide restorations demonstrated unmatched durability with no recorded failures. Layered zirconium oxide restorations, though highly successful overall exhibited limitations under occlusal stresses primarily through chipping. This distinction underscores the significance of material selection and meticulous clinical planning in achieving both long-term survival and functional success of restorations.

## 4. Discussion

This study provides valuable insights into the survival rates and failure modes of zirconium oxide restorations, particularly when comparing monolithic and layered forms. The overall survival rate for zirconium oxide restorations was high, with monolithic restorations achieving a 100% survival rate, while layered restorations exhibited a survival rate of 95.8%. These results align with previous literature suggesting that monolithic zirconium oxide restorations tend to be more durable due to their inherent strength and the absence of additional ceramic layers, which reduces the risk of failure modes such as chipping that are commonly observed in layered restorations [35,36,37].

Monolithic zirconium oxide restorations benefit from their high flexural strength, ranging between 900 and 1200 MPa, which significantly contributes to their favorable performance in clinical settings. This mechanical advantage makes them particularly suitable for posterior restorations, which are exposed to higher mechanical stresses. This finding corroborates studies that have demonstrated a lower incidence of fractures in monolithic zirconium oxide restorations, supporting their use in cases requiring long-term durability [38,39]. In contrast, while layered zirconium oxide restorations offer improved esthetics due to the feldspathic ceramic layer, the results of this study demonstrate a higher failure rate, primarily due to chipping. This observation mirrors prior research, which suggests that layered zirconium oxide is more prone to mechanical failure, particularly when the feldspathic layer is too thick or unsupported [40]. Our findings indicate that when the feldspathic layer exceeds 1.5 mm in thickness, chipping occurs at an accelerated rate, particularly between 24 and 36 months. This supports previous studies that propose thinner ceramic layers may be more prone to failure under functional stress [41].

Another notable finding was the significant difference in survival rates between multi-tooth and single-tooth restorations. Multi-unit restorations are subjected to more evenly distributed occlusal forces, which likely help in reducing the risk of localized stress concentrations that could lead to failure in single-tooth restorations [42]. This raises the point that decisions regarding restoration type should not only consider clinical factors but also the mechanical demands placed on the restoration.

While this study excluded patients with parafunctional habits, such as bruxism, which could have potentially skewed the results towards higher survival rates, it is important to acknowledge the potential impact of such habits. Bruxism and other parafunctional activities are well-known risk factors for restoration failure, and excluding these patients likely contributed to the high survival rate observed in this study [43]. Future research that includes patients with these habits would be essential to further evaluate their effect on the long-term performance of zirconium oxide restorations.

An additional aspect that deserves attention are the esthetic challenges associated with zirconium oxide restorations, particularly in the anterior region. While monolithic zirconium oxide offers excellent mechanical properties, its opacity can be a limiting factor when esthetics is a priority. In these cases, materials such as lithium disilicate, which offer superior translucency, may be preferred, despite their lower mechanical strength [44]. This highlights the need for individualized treatment planning, where both the clinical context and patient preferences must be taken into account when selecting materials for anterior restorations.

The study also examined the role of feldspathic veneers in addressing chipping in layered zirconium oxide restorations. Although feldspathic veneers can enhance both the functional and esthetic outcomes of layered restorations, they may not be suitable for full-contour zirconium oxide restorations due to challenges in achieving proper adhesion. This emphasizes the importance of structural integrity during the design phase of restorations. Failures associated with poorly supported feldspathic layers are not uncommon, and careful attention to optimal layer thickness is critical to prevent future issues [45].

Traditional metal–ceramic restorations are known for their predictable strength, satisfactory esthetic results, and long-term durability in the oral environment [45], but these restorations often require the removal of a significant amount of tooth structure during preparation [17]. Additionally, research suggests that zirconium oxide tends to offer superior fatigue resistance compared to lithium disilicate, underlining the role of substrate stiffness in the mechanical performance of monolithic dental materials [46].

A key factor in this study is the margin preparation and the choice of zirconium oxide as the restorative material. Proper margin adaptation is essential for ensuring a tight seal and long-term restoration stability. Zirconium oxide, with its high stiffness and low thermal expansion, provides excellent marginal fit, making it ideal for precise adaptation. Recent in vitro studies have shown that zirconium oxide crowns exhibit superior marginal accuracy compared to other materials, with values well below the clinically accepted threshold for marginal gaps, making it a reliable choice for posterior restorations [47].

Lastly, the clinical application of zirconium oxide restorations in both single- and multi-unit cases underscores the importance of careful planning and execution. Despite the higher risk of chipping associated with layered restorations, multi-unit zirconium oxide restorations, when designed with appropriate support and occlusal force distribution, showed high durability. In contrast, single-unit restorations, particularly those with layered structures, should be used cautiously due to their higher susceptibility to localized failure [47].

Despite the valuable insights provided by this study, several limitations should be considered when interpreting the results. First, the study was retrospective in nature, which inherently carries the risk of selection bias and limited control over confounding variables. The exclusion of patients with parafunctional habits may limit the generalizability of the findings to the broader patient population where such habits are prevalent. Parafunctional activities, such as bruxism, are well-established risk factors for restoration failure, and their exclusion may have contributed to the higher survival rates observed. Future research should aim to include patients with parafunctional habits to assess their impact on the long-term performance of zirconium oxide restorations, especially in real-world clinical settings. Additionally, the study focused solely on zirconium oxide restorations, so the results may not be directly applicable to other restorative materials. Another limitation is the relatively short duration of follow-up, as the study spanned five years, and longer-term data would be necessary to assess the true durability of these restorations. Furthermore, while complications such as chipping and fracture were explored, other potential issues like wear or esthetic changes over time were not evaluated.

## 5. Conclusions

- This five-year retrospective study evaluated 1143 zirconium oxide restorations: 144 monolithic and 999 layered.
- 42 failures were observed in the layered restorations, while no failures occurred in the monolithic restorations, yielding an overall survival rate of 96.3%.
- The survival rate for layered restorations was 95.8%, compared to 100% for monolithic restorations.
- Failures in layered restorations were primarily caused by chipping when unsupported feldspathic ceramic exceeded 1.5 mm, which can be addressed with feldspathic veneers.
- Monolithic restorations demonstrated superior durability and reliability.
- The study emphasizes the importance of selecting the appropriate zirconium oxide restoration type based on clinical needs, guiding dental professionals toward more informed decisions for long-term success.

## Figures and Tables

**Table 1:** The Case Processing Summary for the Different Levels of Type of Restoration.

Type of Restoration	Total N	N of Events	Censored	
			N	Percent
Layered	999	42	957	95.8%
Monolithic	144	0	144	100.0%
Overall	1143	42	1101	96.3%

**Table 2:** The Case Processing Summary for Zirconium Oxide Restorations in Single vs. Multi-Tooth Cases.

Tooth	Total N	N of Events	Censored	
			N	Percent
Multi-Tooth	108	0	108	100.0%
Single-Tooth	1035	42	993	95.9%
Overall	1143	42	1101	96.3%

**Table 3:** The Case Processing Summary for Maxillary and Mandibular Zirconium Oxide Restorations.

	Total N	N of Events	Censored	
			N	Percent
Mandible	393	15	378	96.2%
Maxilla	750	27	723	96.4%
Overall	1143	42	1101	96.3%

**Table 4:** Restorations Association between Survival Duration in months and Type of Restoration.

	Valid	Mean ± SD	Median (Q1–Q3)
Layered	999	94.29 ± 28.36	88 (75–111)
Monolithic	144	79.71 ± 23.67	68 (68–73)

**Table 5:** Association between Survival Duration in Months and Type of Restoration.

		Cause of Failure/Not Applicable				
Success/ Failure		Chipping of the Feldspathic Ceramic Layer	Coronal Fracture	Coronal Fracture with Root Fracture	Not Applicable	Total
Fail	Count	30	6	6	0	42
	% within row	71.43%	14.29%	14.29%	0.000%	100.000%
Succes	Count	0	0	0	1101	1101
	% within row	0.000 %	0.000 %	0.000 %	100.000%	100.000%
Total	Count	30	6	6	1101	1143
	% within row	2.625 %	0.525 %	0.525 %	96.325%	100.000%

**Table 6:** Comparative Analysis of Causes of Failure in Zirconium Oxide Restorations: Layered vs. Monolithic.

	Valid	Mean ± SD	Median (Q1–Q3)
Multiple Tooth	108	106 ± 21.278	121 (89–121)
Single tooth	1035	91.038 ± 28.491	86 (72–99)
Maxilla	750	96.912 ± 27.199	97(75–121)
Mandible	393	83.939 ± 28.218	77 (68–97)

## Data Availability

The data supporting the reported results in this study are unavailable due to ethical restrictions.
